# A Rare Case of Unicuspid Aortic Valve with Postoperative Heart Block

**DOI:** 10.7759/cureus.5286

**Published:** 2019-07-31

**Authors:** Divya Ravi, Muhammad Siddique Pir, Najam Saqib, Gaurav Patel, Haitham Abughnia

**Affiliations:** 1 Internal Medicine, The Wright Center for Graduate Medical Education, Scranton, USA; 2 Internal Medicine, Wright Center for Graduate Medical Education, Scranton, USA; 3 Cardiology, The Wright Center for Graduate Medical Education, Scranton, USA; 4 Cardiology, The Wright Center for Graduate Medical Education, Clark Summit, USA

**Keywords:** congenital, aortic stenosis, unicuspid aortic valve, pacemaker

## Abstract

Unicuspid aortic valve (UAV) is an extremely rare cause of aortic stenosis, accounting for about 0.02% of the adult population. We present a rare case of UAV in a young woman who presented with dyspnea on exertion. She underwent extensive work-up, including a transthoracic echocardiogram, which was notable for critical aortic stenosis with significant pulmonary hypertension. A subsequent transesophageal echocardiogram confirmed the severe degree of aortic stenosis and revealed the possibility of a UAV. She was referred to a cardiothoracic surgeon and underwent bioprosthetic aortic valve replacement. Intraoperative evaluation confirmed the rare occurrence of a UAV. Postoperative course was complicated by complete heart block necessitating pacemaker placement.

## Introduction

Aortic stenosis is the abnormal narrowing of the aortic valve outlet. It is one of the most common valvular lesions, with an estimated prevalence of 2%-9% in the general adult population above the age of 65 years [1]. Unicuspid aortic valve (UAV) is an extremely rare cause of aortic stenosis, accounting for 0.02% of the adult population [2]. We present a case of UAV in a young woman who eventually underwent successful bioprosthetic aortic valve replacement.

## Case presentation

**Figure 1:**
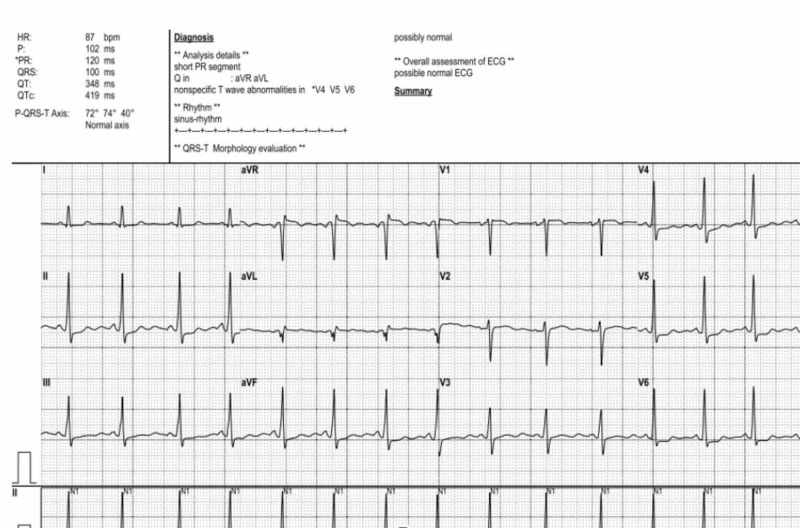
Grossly Unremarkable Electrocardiogram of the Patient

**Figure 2:**
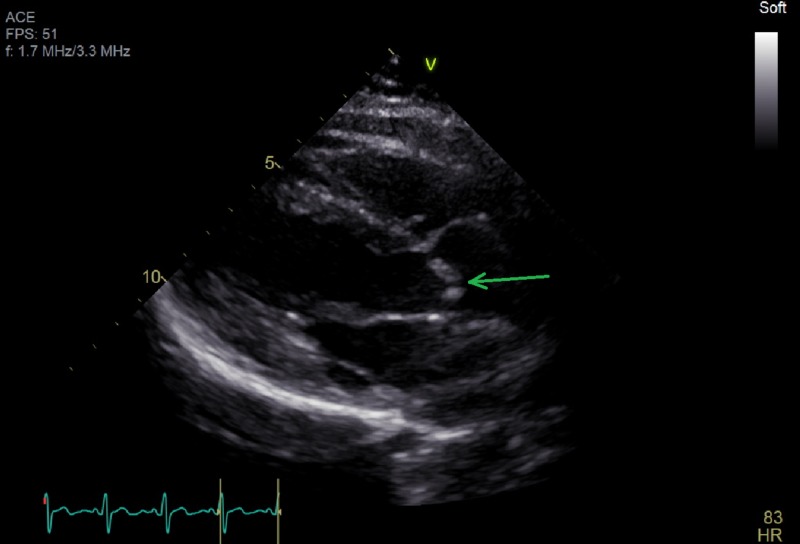
Parasternal Long Axis View Showing Eccentric Closure of Aortic Valve (arrow)

**Figure 3:**
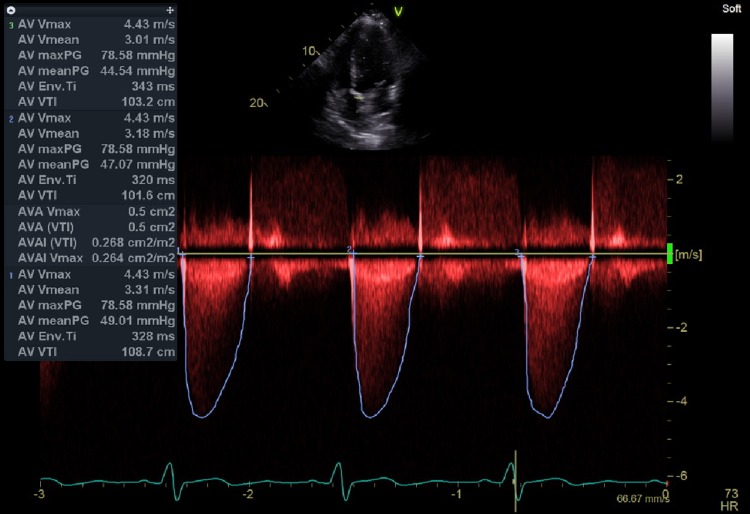
Transthoracic Echocardiogram showing Aortic Valve Area, Peak Velocity and Mean Gradient Consistent with Severe Aortic Stenosis

We report a case of a 28-year-old woman who presented to the office with complaints of progressive exertional dyspnea of three months duration. Of note, she was diagnosed with congenital aortic valve stenosis at birth and underwent balloon valvuloplasty as a neonate. She was followed by a pediatric cardiologist through her childhood and reported no difficulty with moderate-to-strenuous activity as an adolescent. However, as an adult, she spent several years of her life outside the country and was unable to follow up with a cardiologist during that interim period. On return to the States, she re-established care in light of her ongoing symptoms. She reported severe dyspnea on exertion with occasional lightheadedness and palpitations. A review of systems was negative except the aforementioned symptoms. On exam, she had an ejection systolic murmur with a click, best heard at the apex, and radiating to her neck. Electrocardiography (Figure 1) was unremarkable while a transthoracic echocardiogram (TTE) (Figures 2 and 3) was notable for critical aortic stenosis (valve area 0.5 cm^2^, mean gradient 40 mm of Hg) with significant pulmonary hypertension (pulmonary artery pressure 80-84 mm Hg systolic). Both ventricles appeared grossly normal with an estimated ejection fraction 55%. We consulted an adult congenital heart disease specialist and performed a transesophageal echocardiogram (TEE). The TEE confirmed the degree of aortic stenosis and indicated the possibility of a UAV. She was referred to a cardiothoracic surgeon and underwent bioprosthetic aortic valve replacement. The intraoperative evaluation confirmed the rare occurrence of a UAV. However, the postoperative course was complicated by complete heart block with pacemaker placement.

## Discussion

This case report was notable for not only the rare presentation of UAV in a young female patient but also the postoperative complication of complete heart block warranting a pacemaker. The unicuspid aortic valve is a rare congenital anomaly with an annual incidence of less than 0.02% in the general adult population with four times higher prevalence among males [2]. Patients with UAV may present with dyspnea and a systolic murmur from underlying heart failure and aortic stenosis, respectively; this combination, especially in a young patient, must raise suspicion of a UAV. A review by Mookadam et al reported that nearly 41% of the cases manifested with isolated aortic stenosis and 28% had aortic stenosis with or without regurgitation [3]. While a majority of UAV cases are diagnosed on autopsy or pathological examination of the excised valve, the diagnostic modality best suited to clinically diagnose UAV is a three-dimensional TEE [4]. A TTE is unlikely to show enough detail to differentiate a unicuspid from a bicuspid aortic valve. Rarely, the diagnosis is made on intraoperative visualization of the valve [2,4]. As seen in our case, a TTE was only able to identify the presence of aortic stenosis, a follow-up TEE raised suspicion of UAV which was subsequently confirmed during the open surgical procedure.

Another distinguishing feature of this case is the postoperative development of heart block. The mechanism is related to the close anatomical proximity between the aortic valve and conduction system that predisposes to perioperative conduction abnormalities with aortic valve surgeries. While the development of conduction abnormality among patients undergoing isolated aortic valve replacement is relatively significant (up to 1 in 12, in a study by Dawkins et al) [5], we could only identify two other case reports of UAV in the literature that described postoperative complete heart block requiring pacemaker placement [6-7].

## Conclusions

This report describes a rare presentation of aortic stenosis in a young female adult in the setting of a known history of congenital heart disease. Our case adds to the body of literature by highlighting key diagnostic modalities such as transesophageal echocardiogram and intraoperative visualization that can aid in the diagnosis of this rare anomaly. Additionally, this case outlines the possibility of perioperative conduction abnormalities associated with aortic valve surgeries.
